# Diagnostic Radiology: What the Advanced Practitioner Needs to Know

**DOI:** 10.6004/jadpro.2016.7.3.13

**Published:** 2016-04-01

**Authors:** Joseph R. Steele

**Affiliations:** University of Texas MD Anderson Cancer Center, Houston, Texas

Advanced practitioners should use imaging resources wisely by understanding the appropriate use of chest x-ray, CT, and PET/CT, according to Joseph R. Steele, MD, of the University of Texas MD Anderson Cancer Center, Houston, Texas.

At JADPRO Live at APSHO, Dr. Steele described the ABCs of diagnostic radiology, outlining which tests are best for which situations, and which are a misuse of healthcare dollars. It has been estimated that $12 billion is wasted each year on unnecessary imaging (peer60 Report, 2014).

"We need to be responsible stewards of imaging. Think about what you are ordering. Remember that these are very expensive tests that patients often have to pay for themselves," he said. Patients need imaging done, often repeatedly, but clinicians should know what is appropriate. Dr. Steele offered several guidelines for assessing this:

- Ask yourself, what question am I trying to answer? Communicate this to the radiologist.
- Ask next, will this answer change what I am going to do? If not, the test is probably not warranted.
- Generally, start simple: For a cough or fever, for example, order a chest x-ray first. There are, however, situations where a PET scan should be the first test.
- Have previous studies available, if possible, to look for changes vs. baseline. This can be challenging since patients use different imaging centers, often driven by third-party payer requirements. Express the importance of these images and engage the patient as an active part of their own care.

"The most important thing to communicate to the radiologist is the patient’s history," he said. "Tell the radiologist what you hope to find from this study. That way, we can select the right study and tailor the exam and discussion so that the report gives you the information you need."

"No one expects you to always know which test to order," he added. When advanced practitioners have questions, they should always ask. "Radiologists want to be part of the team," he said.

## IONIZING RADIATION

In order to educate patients, it’s good to know the approximate amount of ionizing radiation the various tests emit. "There’s lots in the press about this, and tons of misunderstanding and patient fear," he said.

To put radiation dose into context, he noted that residents of Aspen, Colorado are exposed to about 6 mSv (millisievert) annually. Imaging tests carry the following radiation doses:

- Interventional radiology/cardiac catheterization (dose varies widely; potential for highest dose)
- PET/CT (25 mSv)
- CT of abdomen and pelvis (10–20 mSv)
- Mammography (0.4 mSv)
- Chest x-ray (0.1 mSv)
- MRI (0)
- Ultrasound (0)

"Chest x-rays and mammography have very little radiation," he said. "Hopefully, with this information you can allay your patient’s fears."

## BASICS OF CHEST X-RAY

Dr. Steele’s main advice for interpreting chest x-rays was to "have a standard approach and use it every time."

Most radiologists read a chest x-ray "from inside out," he said, starting with the mediastinum, moving to the lungs, then to the bones, soft tissue, and abdomen.

He emphasized that one needs to "treat the patient, not the x-ray," and advised that "if the imaging study is not jibing with your clinical gut feeling, which is often the case with cancer patients, a CT scan may be warranted."

Cancer patients often have subtle findings and chest x-rays may be too rudimentary to pick them up, he explained.

Masses in the mediastinum may respresent benign or malignant disesase, including primary and metastatic neoplasms. Similarly, the lung may have primary or metastatic disease which is confused with inflammatory disease or infection.

## CATHETER PLACEMENT

Chest x-rays can confirm the position of central venous catheters, as this is institution-specific. Typically, there are policies that say where the catheter should be placed for chemotherapy. Generally, however, for chemotherapy infusion the large central vein is the target. "This means aiming for the superior vena cava or the junction of the superior vena cava and right atrium," Dr. Steele said. Clinicians should understand their facility’s policy and the radiologist’s terminology.

One image may not be sufficient to show placement. "If there is confusion, get a lateral view," he advised. "If it’s very confusing, call the radiologist."

## CT SCANS

Most CT scans in cancer patients are to evaluate the chest, pelvis, and abdomen. They are used for diagnosing and staging, assessing treatment response, and monitoring for recurrence.

Dr. Steele elaborated on liver CT, explaining the need to evaluate all segments with multiphasic imaging. Certain tumors are seen during the arterial phase and others seen more during the venous phase (Figure). A noncontrast CT scan is usually not needed.

**Figure 1:**
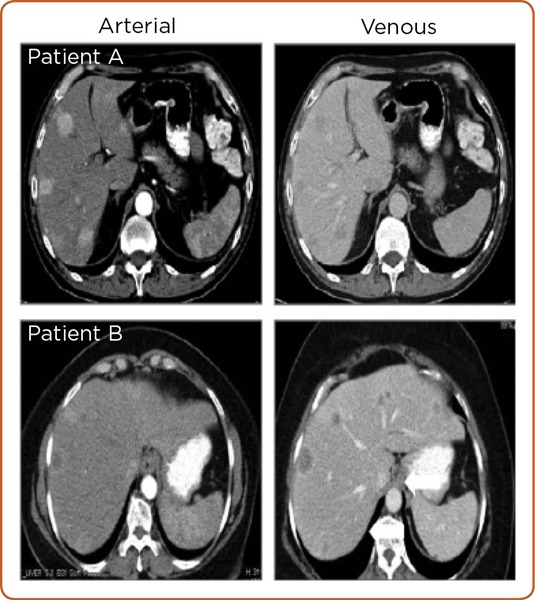
Liver CT. Images courtesy of MD Anderson Cancer Center.

Renal cysts are very common and are described and graded according to Bosniak criteria. Bosniak 1 is a simple cyst that is not worrisome, while Bosniak 4 carries a high likelihood of carcinoma.

Biopsy differentiates between primary renal cancer (RCC) and lymphoma. "The more complex looking, the higher the chance of neoplasm, he said."

Adenopathy in the lymph nodes can represent primary lymphoma or metastatic disease. It is important to "know what you are looking for, and where to look," because it is very difficult to evaluate all nodes.

There are three main areas of focus that correspond to different tumor types: periportal (esophageal, gastric), retroperitoneal (lymphoma, renal, testicular), and pelvis (gynecologic, prostate cancer).

"If you are worried about the liver, get a multiphasic CT," he recommended. This can evaluate for primary tumors or metastatic disease, which is most common in cancers of the colon, breast, and lung.

## PET/CT SCANS

PET/CT vs. conventional CT has several advantages. It is useful under certain conditions, but should not be overused. Medicare now reimburses for only three PET/CT scans per patient. "This is getting to be a problem, as our treatments are getting better. Our patients are now living beyond three PET/CTs," Dr. Steele commented.

In most oncologic applications, PET/CT is thought to be more sensitive and specific than CT alone. They can be very helpful in diagnosis and staging, and can provide feedback regarding response to treatment and possibility of recurrence.

"PET works on the concept that an active tumor will light up more than the surrounding tissue, but that also occurs with infection, inflammation, and other red herrings," he said. "PET/CT is very good at showing response to treatment before CT shows it. Clearly, there are times when it is appropriate and times when it is wasteful."

Dr. Steele recommended the following resources for advanced practitioners who want more information on imaging in cancer:

- www.chestx-ray.com
- *CT for the Nonradiologist* by Rocky Saenz
- *Oncologic Imaging, a Multidisciplinary Approach* by Paul Silverman
- *Diagnostic Imaging: Oncology* by Akram M. Shaaban, Todd M. Blodgett, Paige B. Clark, Marta Heilbrun, and Maryam Rezvani
